# Energy and Nutritional Content of Lunch Menus in Turkish Universities: The Impact on Ecological Footprint

**DOI:** 10.1002/fsn3.70149

**Published:** 2025-04-08

**Authors:** Ozge Yesildemir

**Affiliations:** ^1^ Department of Nutrition and Dietetics, Faculty of Health Sciences Bursa Uludag University Bursa Türkiye

**Keywords:** carbon footprint, food environment, sustainability, university food, water footprint

## Abstract

The aim of this study was to evaluate the relationship between ecological footprints and the nutritional contents of lunch menus in universities. One‐month lunch menus were evaluated from 70 state universities in seven geographic regions in Türkiye. The mean protein, carbohydrate, and fat of the menus were 44.1 ± 2.7 g, 113.7 ± 7.4 g, and 80.9 ± 6.3 g, respectively. Regional differences were observed in the nutrient composition of university lunch menus. The Black Sea region had the highest plant‐based protein, while animal‐based protein was highest in Eastern/Southeastern Anatolia (*p* < 0.05). The Mediterranean region had the highest vitamin B_6_, and sodium content was highest in the Marmara region (*p* < 0.05). The average carbon and water footprints of the menus were 2.26 ± 0.24 CO_2_ eq/kg and 2.14 ± 0.16 m^3^/ton. A positive correlation was observed between menus' energy, saturated fat, vitamin B_12_, sodium, and iron contents and their carbon footprints (*p* < 0.05). Water footprints of menus were positively related to energy, total protein, animal‐based protein, saturated fat, cholesterol, vitamin B_12_, sodium, and iron, and negatively associated with thiamine and zinc (*p* < 0.05). While a one‐unit increase in saturated fat resulted in a 0.829‐unit increase in carbon footprint, menus that increased by a unit in saturated fat increased their water footprint by 0.795 units. When evaluating menus, it is essential to consider nutritional content and environmental impacts together. Universities can design more sustainable and nutritious menus by prioritizing fruits, vegetables, legumes, nuts, seeds, and whole grains while moderately reducing red meat consumption, ultimately lowering ecological footprints and improving students' and staff's dietary quality.

## Graphical abstract

Higher energy, saturated fat, vitamin B_12_, sodium, and iron contents in menus contribute to increased carbon footprints. Water footprints of menus rise with elevated energy, total protein, animal‐based protein, saturated fat, cholesterol, vitamin B_12_, sodium, and iron levels. Menus rich in energy and animal‐based foods have a greater environmental impact, emphasizing the need for sustainable dietary strategies.

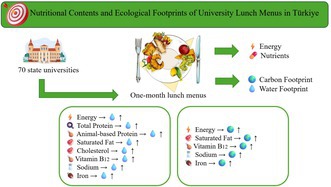

## 1 Introduction

Nutrition is one of the most critical needs for the continuation of human health and well‐being. However, ensuring food security—defined as the ability of individuals to access sufficient, safe, and nutritious food at all times—has become increasingly challenging due to the rapid growth of the world population, climate change, and the depletion of natural resources (Akbari et al. 2022; Wijerathna‐Yapa and Pathirana 2022). Sustainable food production and consumption are essential to addressing these concerns, as reflected in global initiatives such as the Sustainable Development Goals 2030 (Andersson and Hatakka 2023). Shifting toward sustainable dietary patterns can significantly reduce environmental impacts, including carbon and water footprints, while promoting public health (Edalati et al. 2021; Parkin and Attwood 2022; Saleki et al. 2023).

The EAT‐Lancet Commission introduced a universal reference diet emphasizing a high intake of whole grains, vegetables, fruits, legumes, and nuts, with moderate consumption of meat and dairy products and a limited intake of added sugars and fats to promote human and environmental health (Willett et al. 2019). However, Western dietary shifts, characterized by higher animal‐based food consumption, threaten public health by increasing the prevalence of nutrition‐related diseases and raising environmental concerns (Madalı et al. 2021). Moreover, people in many parts of the world experience health problems caused by inadequate and unbalanced nutrition. Balancing nutritional adequacy and sustainability is essential to mitigate these adverse effects (Kemaloglu et al. 2023).

Food system activities directly contribute to greenhouse gas emissions, including carbon dioxide from fossil fuels, fertilizer‐derived nitrous oxide, and methane from livestock farming and rice production. The carbon footprint of a product or service refers to the total greenhouse gas emissions it creates from production to consumption (Xu et al. 2022). The carbon footprint is affected by the food waste generated during the preparation, cooking, and consumption of the meals served in mass catering (Saleki et al. 2023). The water footprint represents the sum of all the water used in a supply chain, comprising blue, green, and gray water. The blue water footprint shows the total fresh surface and groundwater volume needed to produce a commodity. The green water footprint refers to the rainwater consumed by plants and crops. The gray water footprint is the volume of freshwater required to eliminate the pollution caused by product production (Hoekstra et al. 2011).

The mass catering industry in Türkiye needs to start serving healthy and sustainable menus, as in other countries. It is essential to renew the menus serving in mass catering to improve sustainability, reduce negative environmental impacts, promote the conservation of natural resources, reduce food waste, and ensure healthy nutrition (Volanti et al. 2022). Additionally, considering that a lunch meal provides 35%–40% of daily energy intake, it can be essential in promoting healthy and sustainable eating habits for university students and staff (Saleki et al. 2023). Despite the growing awareness of sustainable nutrition, research on the environmental impact of mass catering menus, particularly in Turkish universities, remains limited. Although previous studies have evaluated the ecological footprint of university dining halls, canteens, cafeterias, and restaurants in different countries, they have generally focused on a single institution (Edalati et al. 2021; Hatjiathanassiadou et al. 2019; Saleki et al. 2023; Strasburg and Jahno 2015). However, a comprehensive assessment covering multiple institutions is necessary to gain a more representative understanding of the environmental impact of university food services. Moreover, while it is essential to consider both nutritional content and environmental impact when evaluating menus (Kemaloglu et al. 2023), studies establishing a direct link between these factors remain scarce. Addressing this gap, this study aimed to calculate the carbon and water footprints of lunch menus in Turkish universities and examine their association with energy and nutritional content. By doing so, it seeks to provide a broader perspective on the sustainability of mass catering at the national level and ensure insights into improving sustainability practices in university food services.

## 2 Materials and Methods

The study population included 129 state universities in seven regions of Türkiye. All Turkish universities have reasonably priced food services for students and staff. University dining halls generally serve four‐course meals at lunch daily. This study was conducted considering August 2023 lunch menus published online by universities. August was chosen as the study period because many universities continue to provide regular meal services despite being a summer school period. University dining hall menus are typically planned systematically throughout the year, ensuring consistency in meal offerings. Additionally, August provided access to the most up‐to‐date and comprehensive online university menu records. During the online search, lunch menus from 95 universities were obtained. However, only 70 university lunch menus were evaluated due to incomprehensible dish names and a lack of four‐course meals. The geographical regions where the detected universities are located were determined. Since there were not enough universities from Eastern and Southeastern Anatolia, these two regions were examined together. In addition, a homogeneous distribution was not made when selecting universities in the regions because there are not equal numbers of universities in each area and province. The distribution of the universities by regions included in this study is shown in Table 1. Ethics committee approval was not required, as this study was conducted on publicly available menu lists rather than human subjects. There were no conflicts or restrictions regarding data access, as all menu data were obtained from official university websites, which are publicly accessible.

**TABLE 1:** Distribution of state universities included in the study by regions.

Regions	Total number of state universities	Number of state universities included in the study
Aegean	15	8
Black Sea	20	15
Central Anatolia	27	16
Eastern/Southeastern Anatolia	26	9
Marmara	27	14
Mediterranean	14	8
Total	129	70

### 2.1 Calculations on the Energy and Nutritional Content of Lunch Menus

In this study, the energy and nutrient content of the menus were analyzed using “standard recipes for institutions” in the Nutrition Information System (BeBiS) program (version 9.0). This software is the most widely used tool in calculating the energy and nutrient content of meals in Türkiye. It combines databases of German Nutrient, the US Department of Agriculture, and Turkish Food Composition. Each menu was evaluated as a whole meal. The calculations were performed by entering the ingredient amounts for each meal into BeBiS, which then automatically derived the energy and nutrient values based on the food composition data. The program applies standard conversion factors and cooking yield adjustments when necessary. The final energy and nutrient values for each menu were obtained by summing the contributions of all ingredients.

### 2.2 Calculations on the Carbon Footprint of Lunch Menus

There are different methodologies in the literature regarding calculating the carbon footprints of food products; however, the life cycle assessment approach is generally used. There is currently no nationwide data available on carbon footprint values for various foods produced in Türkiye. Thus, data from a systematic review by Clune et al. (2017) were used to calculate carbon footprint values. The specific carbon footprint factors using this study are provided in Table S1. To calculate the carbon footprint of each menu, the following formula was applied:

$$\text{CFmenu}=\sum \left(\text{gfood}\;\times \;\text{CFfactor}\right)$$

where:

- CFmenu is the total carbon footprint of the menu (CO_2_ eq/g).
- gfood is the amount of each food in grams.
- CFfactor is the carbon footprint factor of the food (kg CO_2_ eq/g).

The food grams of each menu were multiplied by their carbon footprint factors, and finally, the total carbon footprint of the menu was calculated. The carbon footprints of the menus were given in CO_2_ eq/kg.

### 2.3 Calculations on the Water Footprint of Lunch Menus

Various methodologies have been developed to evaluate the impacts of food production on water resources, including water footprint assessment (Hoekstra et al. 2011) and water‐focused life cycle assessment (Boulay et al. 2013). Since the water footprint assessment created by the Water Footprint Network is more detailed and has been used in many nutrition‐related studies (Vanham et al. 2016, 2013), the same approach was preferred in this study. The water footprints of meals were calculated using data from Hoekstra et al. (2011); Mekonnen and Hoekstra (2012), who provided the weighted mean water footprint factors of many animal and agricultural products for different countries, including Türkiye. The specific water footprint factors using this study are provided in Table S2. To calculate the water footprint for each menu, the following formula was applied:

$$\text{WFmenu}=\sum \left(\text{gfood}\;\times \;\text{WFfactor}\right)$$

where:

- WFmenu is the total water footprint of the menu (m^3^/g).
- gfood is the amount of each food in grams.
- WFfactor is the carbon footprint factor of the food (m^3^ per g of food).

The food gram amounts of each menu were multiplied by their water footprint factors (m^3^/g), and finally, the total water footprint of the menu was calculated. All three components of water footprints (blue, green, and gray) were also computed for all menus. The final results were then converted to m^3^ per ton of menu served.

### 2.4 Statistical Analysis

Statistical analyses were performed using IBM SPSS Statistics Version 23 (IBM Inc., Armonk, NY, USA). Data were presented as the mean ± standard deviation (SD), regression coefficient with 95% confidence intervals (CIs), or r square (*R* ^2^) as appropriate. The Shapiro–Wilk test was used to assess normality. One‐way ANOVA analysis was applied to investigate whether there were differences in the menus' energy, macro, and micronutrient contents by region. The Bonferroni test was used as a multiple comparison test. Simple linear regression was applied to identify the predictors of carbon and water footprint. Furthermore, multiple linear regression analysis was performed to investigate the effect of some macronutrients on the carbon and water footprint. Total protein, plant‐based protein, animal‐based protein, total carbohydrate, total fat, saturated fat, and dietary fiber were selected as predictors. Results were evaluated in a 95% confidence interval and statistically at a *p* < 0.05 significance level.

## 3 Results

The mean energy and nutrient content of lunch menus in universities are shown in Table 2. The average energy content of all university menus was 1305.8 ± 66.4 kcal, and there is no statistically significant difference between regions (*p* > 0.05). The mean protein, carbohydrate, and fat of the menus were 44.1 ± 2.7 g (12.5%), 113.7 ± 7.4 g (34.8%), and 80.9 ± 6.3 g (52.7%), respectively. We found statistically significant differences in menus' plant‐ and animal‐based protein contents between regions (*p* < 0.05). The amount of plant‐based protein in the menus served at universities in the Black Sea region (17.2 ± 0.9 g) was higher than that in the Aegean (15.5 ± 1.2 g) and Mediterranean (15.6 ± 1.0 g) regions. The animal‐based protein was highest in the menus of Eastern/Southeastern Anatolia regions (31.5 ± 1.5 g). A significant difference in the dietary fiber content of menus was found between the Black Sea region (12.4 ± 0.8 g) and Eastern/Southeastern Anatolia region (11.3 ± 0.5 g) (*p* < 0.05). The mean vitamin B_6_ content of lunch menus was 3.5 ± 1.9 mg, and the menus of universities in the Mediterranean region had the highest vitamin B_6_ content (5.8 ± 3.1 mg) (*p* < 0.05). The highest sodium and zinc content were on the menus of universities in the Marmara and Mediterranean regions, respectively (*p* < 0.05) (Table 2).

**TABLE 2:** Energy and nutritional content of lunch menus in universities according to regions.

Energy and Nutrients	Aegean (*n*: 8)	Black Sea (*n*: 15)	Central Anatolia (*n*: 16)	Eastern/Southeastern Anatolia (*n*: 9)	Marmara (*n*: 14)	Mediterranean (*n*: 8)	Total (*n*: 70)	*p*
	Mean ± SD	Mean ± SD	Mean ± SD	Mean ± SD	Mean ± SD	Mean ± SD	Mean ± SD	
Energy (kcal)	1293.7 ± 74.4	1327.5 ± 49.0	1295.7 ± 42.4	1280.0 ± 70.8	1334.8 ± 86.9	1275.5 ± 67.4	1305.8 ± 66.4	0.157
Protein (g)	43.8 ± 2.7	44.5 ± 2.2	43.0 ± 2.2	45.4 ± 2.2	44.7 ± 3.2	42.8 ± 3.3	44.1 ± 2.7	0.167
Plant‐based protein (g)	15.5 ± 1.2^a^	17.2 ± 0.9^b^	16.5 ± 1.4^ab^	15.8 ± 1.2^ab^	16.5 ± 0.9^ab^	15.6 ± 1.0^a^	16.4 ± 1.2	**0.004** *
Animal‐based protein (g)	29.8 ± 2.7^ab^	28.6 ± 1.9^ab^	27.6 ± 3.0^a^	31.5 ± 1.5^b^	29.4 ± 3.0^ab^	29.0 ± 2.8^ab^	29.1 ± 2.8	**0.018** *
Carbohydrate (g)	114.3 ± 6.5	114.8 ± 6.6	113.0 ± 5.6	109.4 ± 6.9	115.3 ± 8.4	114.2 ± 10.9	113.7 ± 7.4	0.513
Fat (g)	81.7 ± 5.2	80.3 ± 6.5	79.1 ± 5.4	78.2 ± 5.6	82.9 ± 6.9	84.0 ± 7.9	80.9 ± 6.3	0.272
Saturated fat (g)	16.1 ± 1.6	16.3 ± 1.1	16.1 ± 1.0	16.0 ± 1.1	16.9 ± 1.6	15.9 ± 1.4	16.3 ± 1.3	0.516
Omega‐3 (g)	1.1 ± 0.2	1.1 ± 0.2	1.1 ± 0.1	1.2 ± 0.1	1.1 ± 0.2	1.1 ± 0.2	1.1 ± 0.1	0.368
Cholesterol (mg)	119.6 ± 17.4	114.6 ± 12.3	109.8 ± 14.4	120.3 ± 9.0	120.9 ± 16.2	113.9 ± 26.8	116.0 ± 16.0	0.424
Dietary fiber (g)	12.2 ± 1.0^ab^	12.4 ± 0.8^a^	12.3 ± 1.0^ab^	11.3 ± 0.5^b^	11.9 ± 0.6^ab^	12.0 ± 0.8^ab^	12.1 ± 0.9	**0.048** *
Vitamin A (mcg)	511.2 ± 109.4	493.7 ± 110.9	538.0 ± 128.4	487.4 ± 111.7	516.8 ± 156.4	532.7 ± 159.5	514.0 ± 127.6	0.965
Vitamin E (mcg)	33.0 ± 3.3	35.4 ± 3.8	33.9 ± 3.8	32.9 ± 3.4	35.3 ± 3.1	32.1 ± 2.7	34.1 ± 3.5	0.141
Thiamine (mg)	0.6 ± 0.1	0.6 ± 0.0	0.6 ± 0.4	0.6 ± 0.0	0.6 ± 0.0	0.6 ± 0.1	0.6 ± 0.0	0.130
Riboflavin (mg)	1.2 ± 0.3	0.9 ± 0.2	1.0 ± 0.2	1.0 ± 0.3	0.9 ± 0.2	1.2 ± 0.3	1.0 ± 0.2	0.053
Vitamin B_6_ (mg)	4.4 ± 1.8^ab^	2.7 ± 1.0^a^	3.0 ± 1.2^a^	3.2 ± 1.9^a^	3.4 ± 1.4^a^	5.8 ± 3.1^b^	3.5 ± 1.9	**0.039** *
Folic acid (mcg)	166.8 ± 13.7	167.6 ± 14.1	161.1 ± 18.6	151.4 ± 10.5	159.6 ± 10.2	159.1 ± 8.0	161.4 ± 14.1	0.101
Vitamin B_12_ (mcg)	3.0 ± 0.4	2.7 ± 0.3	2.8 ± 0.3	2.6 ± 0.6	3.0 ± 0.3	2.9 ± 0.3	2.8 ± 0.4	0.124
Vitamin C (mg)	84.4 ± 21.5	73.1 ± 12.0	72.9 ± 10.2	66.9 ± 18.3	74.0 ± 12.6	86.1 ± 22.4	75.2 ± 15.9	0.503
Sodium (mg)	1759.9 ± 273.1^a^	2046.7 ± 194.4^ab^	2038.3 ± 187.8^ab^	2112.5 ± 252.5^b^	2154.1 ± 252.2^b^	2102.4 ± 236.5^ab^	2048.3 ± 246.5	**0.008** *
Potassium (mg)	1690.3 ± 139.5	1608.9 ± 105.0	1596.4 ± 104.7	1563.2 ± 73.8	1606.3 ± 111.9	1599.5 ± 77.0	1607.9 ± 106.2	0.248
Calcium (mg)	260.9 ± 17.5	251.4 ± 30.3	244.2 ± 17.7	228.5 ± 21.4	266.4 ± 39.4	231.9 ± 36.0	248.7 ± 30.6	0.060
Magnesium (mg)	206.9 ± 19.6	193.1 ± 9.4	194.9 ± 19.1	193.7 ± 20.0	200.8 ± 19.5	221.2 ± 29.0	199.9 ± 20.5	0.073
Iron (mg)	7.1 ± 0.6	7.1 ± 0.4	7.0 ± 0.5	6.9 ± 0.4	7.0 ± 0.5	7.2 ± 0.4	7.0 ± 0.5	0.698
Zinc (mg)	10.7 ± 2.4^ab^	8.3 ± 1.5^b^	8.7 ± 1.8^b^	8.8 ± 2.8^b^	9.3 ± 1.9^b^	13.0 ± 4.4^a^	9.5 ± 2.7	**0.019** *

*Note:* The superscripts (^a,b^) show the results of pairwise comparisons between countries; values with unlike letters were significantly different among groups.

* The bold values indicate statistically significant values (*p* < 0.05).

**FIGURE 1:**
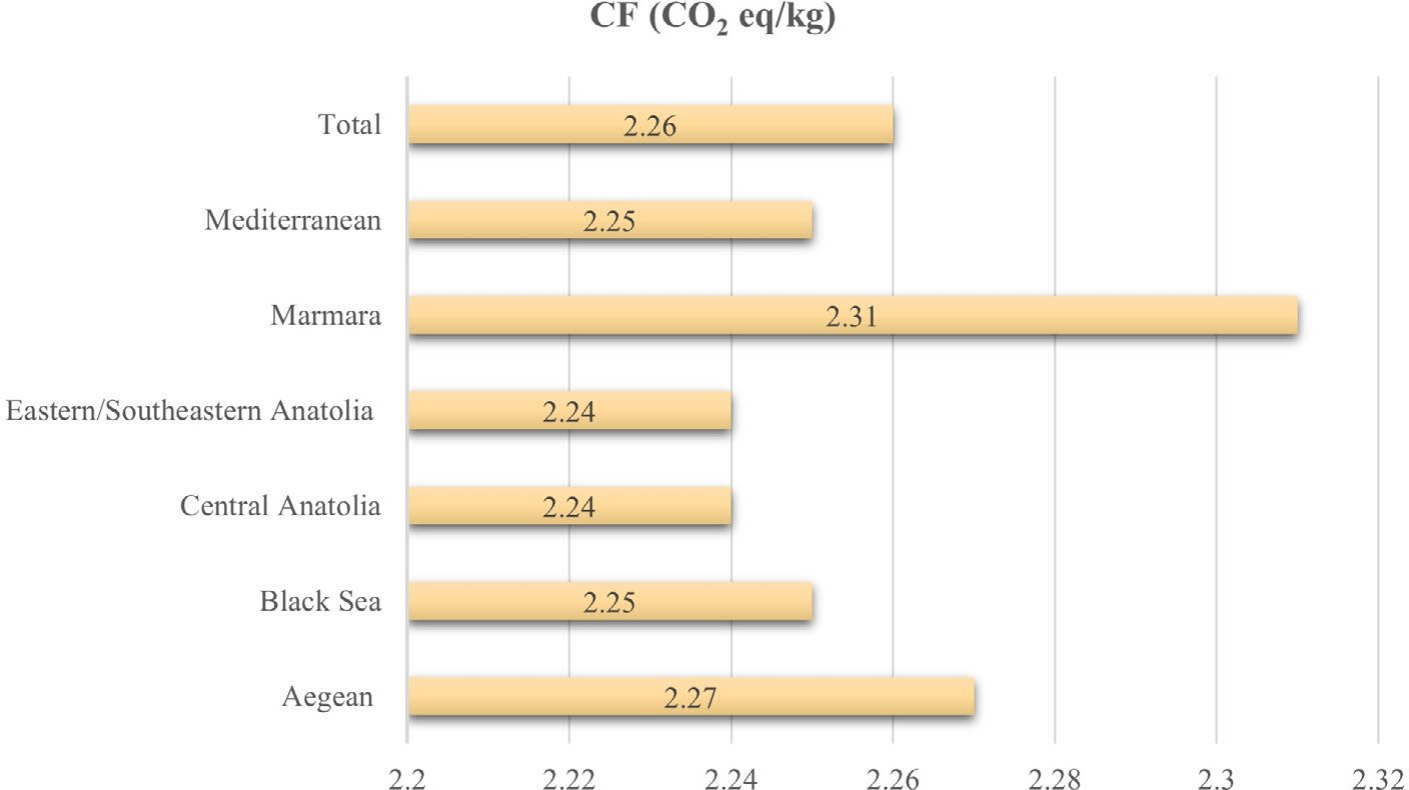
Carbon footprint (CF) of lunch menus in universities according to regions (CO_2_ eq/kg).

**FIGURE 2:**
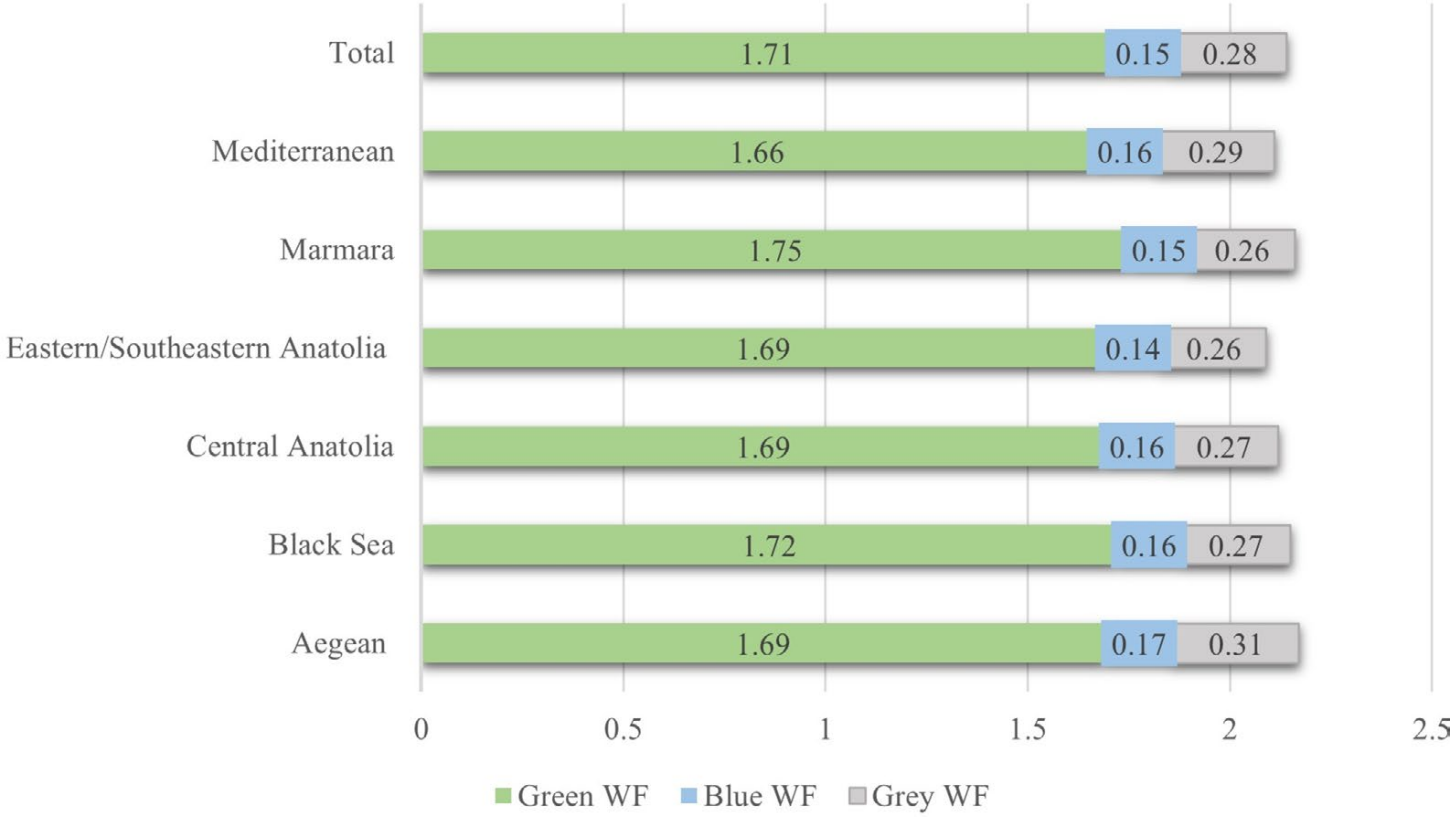
Total (green, blue and gray) water footprint (WF) of lunch menus in universities according to regions (m^3^/ton).

The mean carbon footprint of lunch menus was 2.26 ± 0.24 CO_2_ eq/kg, and no statistically significant difference exists between regions (*p* = 0.982) (Figure 1). We calculated the mean total water footprint as 2.14 ± 0.16 m^3^/ton. The average green, blue, and gray water footprints were 1.71 ± 0.13 m^3^/ton, 0.15 ± 0.02 m^3^/ton, and 0.28 ± 0.07 m^3^/ton, respectively. There is no statistically significant difference in green (*p* = 0.689), blue (*p* = 0.106), gray (*p* = 0.514), and total water footprint (*p* = 0.891) between regions (Figure 2).

Table 3 shows the relationship between the ecological footprints of menus and their energy and nutrient contents. A positive relationship was found between the carbon footprints of menus and their energy, saturated fat, vitamin B_12_, sodium, and iron contents (*p* < 0.05). Water footprint values were positively associated with energy, total protein, animal‐based protein, saturated fat, cholesterol, vitamin B_12_, sodium, and iron. At the same time, they were negatively related to thiamine, vitamin B_6_, and zinc contents (*p* < 0.05) (Table 3).

**TABLE 3:** Association between ecological footprints and nutrients.

Variables	Carbon footprint					Water footprint				
	*β*	*t*	95% CI	*R* ^2^	*p*	*β*	*t*	95% CI	*R* ^2^	*p*
Energy (kcal)	0.239	2.028	0.000; 0.002	0.057	**0.046**	0.567	5.675	0.001; 0.002	0.321	< **0.001**
Protein (g)	−0.030	−0.246	−0.025; 0.019	0.001	0.807	0.343	3.007	0.006; 0.030	0.117	**0.004**
Plant‐based protein (g)	−0.206	−1.732	−0.089; 0.006	0.042	0.088	0.042	0.350	−0.023; 0.033	0.002	0.727
Animal‐based protein (g)	0.096	0.792	−0.13; 0.029	0.009	0.431	0.270	2.315	0.002; 0.025	0.073	**0.024**
Carbohydrate (g)	−0.030	−0.248	−0.009; 0.007	0.001	0.805	0.193	1.619	−0.001; 0.008	0.037	0.110
Fat (g)	0.152	1.264	−0.003; 0.015	0.023	0.210	0.193	1.625	−0.001; 0.009	0.037	0.109
Saturated fat (g)	0.640	6.861	0.085; 0.154	0.409	< **0.001**	0.714	8.419	0.059; 0.095	0.510	< **0.001**
Omega‐3 (g)	0.286	2.464	0.088, 0.837	0.082	0.116	0.163	1.364	−0.070; 0.373	0.027	0.177
Cholesterol (mg)	0.165	1.379	−0.001; 0.006	0.027	0.173	0.432	3.947	0.002; 0.006	0.186	< **0.001**
Dietary fiber (g)	−0.152	−1.266	−0.109; 0.024	0.023	0.210	−0.086	−0.713	−0.053; 0.025	0.007	0.478
Vitamin A (mcg)	0.128	1.064	0.000; 0.001	0.016	0.291	0.011	0.092	0.000; 0.000	0.000	0.927
Vitamin E (mcg)	−0.051	−0.417	−0.020; 0.013	0.003	0.678	0.198	1.665	−0.002; 0.017	0.039	0.101
Thiamine (mg)	−0.138	−1.150	−2.104; 0.565	0.019	0.254	−0.315	−2.736	−1.744; −0.273	0.099	**0.008**
Riboflavin (mg)	−0.063	−0.518	−0.296; 0.174	0.004	0.606	−0.195	−1.636	−0.242; 0.024	0.038	0.106
Vitamin B_6_ (mg)	−0.177	−1.486	−0.054; 0.008	0.031	0.142	−0.371	−3.293	−0.044; −0.011	0.138	0.202
Folic acid (mcg)	−0.074	−0.615	−0.005; 0.003	0.006	0.541	−0.045	−0.373	−0.003; 0.002	0.002	0.710
Vitamin B_12_ (mcg)	0.689	7.831	0.332; 0.559	0.474	< **0.001**	0.432	3.953	0.080; 0.242	0.187	< **0.001**
Vitamin C (mg)	0.067	0.553	−0.003; 0.005	0.004	0.582	−0.196	−1.649	−0.004; 0.000	0.038	0.104
Sodium (mg)	0.334	2.925	0.000; 0.001	0.112	**0.005**	0.356	3.144	0.000; 0.000	0.127	**0.002**
Potassium (mg)	0.198	1.666	0.000; 0.001	0.039	0.100	0.160	1.334	0.000; 0.001	0.025	0.187
Calcium (mg)	0.154	1.282	−0.001; 0.003	0.024	0.204	0.218	1.844	0.000; 0.002	0.048	0.069
Magnesium (mg)	−0.094	−0.782	−0.004; 0.002	0.009	0.437	−0.171	−1.429	−0.003; 0.000	0.029	0.158
Iron (mg)	0.288	2.478	0.029; 0.272	0.083	**0.016**	0.305	2.641	0.022; 0.161	0.083	**0.010**
Zinc (mg)	−0.092	−0.765	−0.030; 0.013	0.009	0.447	−0.293	−2.530	−0.027; −0.003	0.086	**0.014**

*Note: β* refers to the regression coefficient that demonstrated the change in carbon and water footprint change in the variables. The bold values indicate statistically significant values (*p* < 0.05).

Multiple regression was run to predict carbon footprint from total protein, plant‐based protein, animal‐based protein, total carbohydrate, total fat, saturated fat, and dietary fiber. This resulted in a significant model, *F*_(7,62)_ = 10.426, *p* < 0.001. It was found that 48.9% ($R_{\text{adjusted}}^{2}$ = 0.489) of the variance in the carbon footprint variable was explained by independent variables. The individual predictors were examined further and indicated that total fat (*t* = −2.776, *p* < 0.05) and saturated fat (*t* = 7.885, *p* < 0.001) were significant predictors. A one‐unit increase in total fat resulted in a −0.296 unit (95% CI: −0.019; −0.003) decrease in carbon footprint. Moreover, a one‐unit increase in saturated fat resulted in a 0.829‐unit (95% CI: 0.116; 0.194) increase in carbon footprint (Table 4).

**TABLE 4:** Multiple linear regression analysis of carbon footprint‐related factors.

Variables	*B*	SE	*β*	95% CI	*t*	*p*	$R_{\text{Adjusted}}^{2}$
Total protein (g)	−0.039	0.033	−0.430	−0.104; 0.027	−1.186	0.240	0.489
Plant‐based protein (g)	−0.024	0.047	−0.120	−0.119; 0.071	−0.510	0.612	
Animal‐based protein (g)	0.021	0.035	0.243	−0.048; 0.090	0.614	0.542	
Total carbohydrate (g)	0.004	0.003	0.128	−0.003; 0.011	1.250	0.216	
Total fat (g)	−0.011	0.004	−0.296	−0.019; −0.003	−2.776	**0.007**	
Saturated fat (g)	0.155	0.020	0.829	0.116; 0.194	7.885	< **0.001**	
Dietary fiber (g)	0.002	0.038	0.008	−0.073; 0.078	0.062	0.951	

*Note: β* refers to the regression coefficient that demonstrated the change in water footprint in the variables. The bold values indicate statistically significant values (*p* < 0.05).

When the factors that could affect the water footprint (total protein, plant‐based protein, animal‐based protein, total carbohydrate, total fat, saturated fat, and dietary fiber) were evaluated with multiple regression analysis, the model was important ($R_{\text{adjusted}}^{2}$ = 0.559, *p* < 0.001). It was determined that total protein, plant‐based protein, animal‐based protein, total carbohydrate, and dietary fiber did not affect the water footprint (*p* > 0.05). In contrast, total fat (*p* < 0.05) and saturated fat (*p* < 0.001) affected the model. Lunch menus that increased by one unit in total fat decreased their water footprint by −0.229 units (95% CI: −0.009; −0.001). On the contrary, menus that increased by a unit in saturated fat increased their water footprint by 0.795 units (95% CI: 0.064; 0.106) (Table 5).

**TABLE 5:** Multiple linear regression analysis of water footprint‐related factors.

Variables	*B*	SE	*β*	95% CI	*t*	*p*	$R_{\text{Adjusted}}^{2}$
Total protein (g)	0.020	0.017	0.378	−0.015; 0.055	1.124	0.265	0.559
Plant‐based protein (g)	−0.024	0.025	−0.206	−0.074; 0.027	−0.941	0.350	
Animal‐based protein (g)	−0.013	0.018	−0.267	−0.050; 0.023	−0.727	0.470	
Total carbohydrate (g)	0.003	0.002	0.171	0.000; 0.007	1.793	0.078	
Total fat (g)	−0.005	0.002	−0.229	−0.009; −0.001	−2.311	**0.024**	
Saturated fat (g)	0.085	0.010	0.795	0.064; 0.106	8.145	< **0.001**	
Dietary fiber (g)	0.002	0.020	0.015	−0.038; 0.043	0.119	0.905	

*Note: β* refers to the regression coefficient that demonstrated the change in water footprint in the variables. The bold values indicate statistically significant values (*p* < 0.05).

## 4 Discussion

To the authors' knowledge, this is one of a limited number of studies to evaluate lunch menus in all accessible universities in a country regarding sustainable nutrition principles. Moreover, this study compared university lunch menus' energy and nutrient contents with their ecological footprints. The current study found that menus' energy, saturated fat, vitamin B_12_, sodium, and iron content affected their ecological footprints. This showed that menus containing high‐energy and rich in animal‐based foods had a higher harmful environmental impact.

In university settings, students and staff typically spend a substantial amount of time at the institution, 5–30 h a week, or even more, over many years. This brings a responsibility to universities to provide a food environment that will enable students and staff to make healthier and more sustainable food choices (Martinez‐Perez et al. 2022). Students generally prefer university dining halls, especially for lunch, for various reasons such as the remote location of campuses, busy schedules, and limited financial means (Zainol and Seladorai 2016). University dining halls are unique environments to promote healthy and sustainable nutrition, as they strongly influence the nutritional behavior of students (Saleki et al. 2023).

Improving knowledge and behaviors on dietary behaviors in the college‐age population is critical to designing and supporting campus healthy eating campaigns (Sogari et al. 2018). It is emphasized that university campus dining environments can directly affect students' energy and nutrient consumption. Therefore, more attention should be paid to serving healthy and balanced meals in university dining halls (Whatnall et al. 2021). Furthermore, since the dietary habits of university students are characterized by a higher intake of snacks, fast foods, French fries, cakes, pies, and carbonated beverages, and a lower intake of fruits and vegetables, it is even more critical to provide adequate and balanced menus in universities (Bernardo et al. 2017). The National Menu Planning and Implementation Guide for Mass Nutrition Systems (Mass Consumption Places) was published in 2020 to standardize the energy and nutritional content of menus served in mass catering institutions. This indicates that lunch menus should provide 2/5 of the daily needs of adults. The range of energy in lunch menus is recommended to vary between 640 kcal and 880 kcal (Beyhan et al. 2020). However, the average energy content of lunch menus from all universities in this study was 1305.8 ± 66.4 kcal (Table 2), significantly exceeding the recommended range. Similarly, in a study conducted at a private university in Türkiye, the mean energy was 1695.0 ± 650.0 kcal (Saleki et al. 2023). These findings indicate that university menus may contribute to excessive calorie intake among students, which could have long‐term health implications. Regarding macronutrient distribution, the current guideline recommends that 20%–35% of total energy comes from fat (Beyhan et al. 2020). However, in this study, university menus contained approximately 53% of energy from fat, exceeding the recommended upper limit. Additionally, the protein and carbohydrate contents (44.1 ± 2.7 g and 113.7 ± 7.4 g, respectively) were nearly twice the recommended amounts, suggesting an excessive protein intake and an imbalanced carbohydrate‐to‐protein ratio. According to Türkiye's Dietary Guidelines (TUBER), a balanced diet should include appropriate energy and macronutrient distribution to support overall health and prevent noncommunicable diseases. The recommended proportions of carbohydrate, protein, and fat in total energy intake are 45%–60%, 10%–20%, and 20%–35%, respectively (Ministry of Health of the Republic of Türkiye 2022). In our study, however, the proportions are quite different, with protein contributing 13%, carbohydrates 34%, and fats 53%. This suggests a deviation from the general dietary guidelines, indicating a need for dietary adjustments in university menus. The observed macronutrient imbalances suggest that university meals may not align with these dietary recommendations, which could affect students' long‐term health. In a study conducted in a university canteen in Croatia, the mean energy, protein, carbohydrate, and fat content of a meal were found to be 847.6 ± 399.4 kcal, 42.1 ± 14.2 g, 72.2 ± 32.8 g, and 42.3 ± 29.3 g, respectively (Lachat et al. 2009). The average energy, protein, and carbohydrate content of the lunch menu served at a public university in Türkiye were 1039.8 kcal, 30.7 g, and 80.8 g, respectively (Ozcicek‐Dolekoglu and Var 2019). The later contrary study showed that energy (539.0 ± 128.6 kcal), protein (17.9 ± 6.0 g), and fat content (13.7 ± 7.6 g) of a meal in Indonesian university canteens were below the recommendations (Sakai et al. 2022). In the current study, all micronutrients except calcium were above the recommendations. While a minimum of 380–400 mg of calcium must be provided at lunch (Beyhan et al. 2020), this value was 248.7 ± 30.6 in this study (Table 2). The insufficient amount of calcium indicates that more dairy products should have been included in the menus. Overall, it is clear that university lunch menus exceed the recommended levels of energy and many micronutrients. Although adequate nutrient intake is essential for maintaining health, excessive intake of certain micronutrients may pose health risks. For example, excessive sodium intake is associated with an increased risk of hypertension and cardiovascular diseases, while high levels of certain fat‐soluble vitamins, such as vitamin A, can lead to toxicity (Pike and Zlotkin 2019). Thus, the potential health implications of these excesses should be carefully evaluated in future research. However, actual food consumption is likely lower than that of foods served. It is stated that food waste is high in the university dining halls, where nearly 7%–14% of the meals are not eaten (Ozcicek‐Dolekoglu and Var 2019). Therefore, further studies considering plate waste will provide more accurate results regarding actual nutrient intake. Nevertheless, our results revealed that menu planning needs optimization.

Türkiye comprises seven regions with different dietary habits (Ceylan et al. 2019). Geographical, ecological, cultural, and economic features shape the eating habits of that region (Gokce and Gunay 2017). Differences in food preferences observed locally in these regions are also reflected in university menus. The food culture of the Aegean region, which is shaped by the region's proximity to the coast and historical connections with ancient Mediterranean civilizations, generally focuses on olive oil dishes, vegetable dishes, fish, and fruit consumption. Although regional cuisine is usually based on light foods, many famous deep‐fried dishes and desserts exist (Ceylan et al. 2019). Fish, corn, dishes made from corn flour, kale, fresh and dried beans, potatoes, and rice are the most consumed foods in the Black Sea region (Ceylan et al. 2019; Gokce and Gunay 2017). The high plant‐based protein consumption in the Black Sea region can be attributed to the fertility of the land and the diversity of agricultural activities in the area. Additionally, the region's climatic conditions support the widespread consumption of herbs, vegetables, and legumes. Wheat products are mainly consumed in the Central Anatolia region, which is considered the grain mill of Türkiye. Livestock farming throughout the region also increases the variety of meat dishes (Gokce and Gunay 2017). Eastern/Southeastern Anatolia regions are known for their meat dishes, pitas, and sherbet desserts. Milk and dairy products are also essential; meat dishes are often cooked with yogurt (Ceylan et al. 2019; Gokce and Gunay 2017). The higher meat consumption in these regions reflects traditional livestock farming practices deeply embedded in the region's economy and culture. These agricultural practices and their economic impact are central to understanding the higher meat consumption. Furthermore, historical and cultural factors play an essential role in shaping the dietary habits of the region where meat and dairy products are commonly used (Gokce and Gunay 2017). The Marmara region has a somewhat mixed nutritional pattern. Although the general dietary habits in the Mediterranean region are based on grains, olive oil, vegetables, fruits, seafood, and dairy products, there are differences in the east and west of the area. Meat and spicy and fatty dishes are consumed more in the eastern region (Ceylan et al. 2019). In this study, the amount of plant‐based protein was high in the Black Sea region, where vegetables and legumes are frequently consumed. On the other hand, a low amount of plant‐based protein in the Aegean and Mediterranean regions, where the Mediterranean type of diet is most common, shows a lack of legume dishes on the menus. Animal‐based protein was higher in the Eastern/Southeastern Anatolia regions, which are famous for meat dishes. In support of this, the dietary fiber in the Black Sea region was higher than in the Eastern/Southeastern Anatolia region (Table 2).

Nutrition is directly related to health and the environment, and special attention has been paid to this issue in recent years. The food industry is one of the main drivers of global environmental change. For this reason, besides providing adequate and balanced food services in institutions such as universities, hospitals, schools, and nursing homes, it is necessary to consider these menus' carbon and water footprints (Aytekin‐Sahin et al. 2023). Studies in the literature have evaluated the menus of university dining halls, canteens, cafeterias, and restaurants in different countries regarding ecological footprint; however, they have generally focused on only one institution (Edalati et al. 2021; Hatjiathanassiadou et al. 2019; Saleki et al. 2023; Strasburg and Jahno 2015). Our study is critical in evaluating the menus of all accessible universities in Türkiye. The mean carbon and water footprints of university lunch menus were 2.26 ± 0.24 CO_2_ eq/kg and 2.14 ± 0.16 m^3^/ton, respectively, in this study (Table 2). The December lunch menu in a Turkish private university had a carbon footprint value of 3.4 ± 1.3 CO_2_ eq/kg. In the same study, it was reported that the water footprint value was 3.8 ± 1.1 m^3^/ton (Saleki et al. 2023). A study conducted in residential dining halls at a university setting in the USA found an estimated average carbon footprint per meal served of 2.26 CO_2_ eq/kg. The mean daily carbon footprint per person was calculated at approximately 4.52–6.78 CO_2_ eq/kg, assuming that students enter the dining halls two to three times Daily (Lambrecht et al. 2023). In comparison, the carbon footprint of the average US diet is 4.72 CO_2_ eq/kg per person per day (Heller et al. 2018). This suggests that per capita carbon footprints related to food consumption at universities are likely higher than the average US diet (Lambrecht et al. 2023). Similarly, in a study using data from the Türkiye Nutrition and Health Survey 2017, the dietary carbon footprint was 3.21 ± 2.07 CO_2_ eq/kg/person/day, and the dietary total water footprint was 2.83 ± 1.38 m^3^/person/day (Ilhan et al. 2023). This reveals that the per‐person ecological footprints associated with food consumption at universities are higher than the average Turkish diet. However, caution should be taken when interpreting these estimates, given that data in this study were obtained from menus and thus may overestimate intake compared to diet recall methods.

When evaluating menus, it is essential to consider nutritional content and environmental impacts together (Kemaloglu et al. 2023). This study determined that their carbon footprints also increase as the menus' energy, saturated fat, vitamin B_12_, sodium, and iron levels increase. The relationship between these nutrients and environmental impacts can be better understood through the production processes. Saturated fat, often found in animal‐based products, is directly linked to higher carbon footprint values due to the intensive farming practices required for livestock (Clune 2019). Similarly, vitamin B_12_, which is primarily sourced from animal‐based foods, contributes to the environmental burden because the production of animal‐derived products requires significant resources and energy (González‐García et al. 2020). Water footprint values were positively related to energy, total protein, animal‐based protein, saturated fat, cholesterol, vitamin B_12_, sodium, and iron (Table 3). These nutrients are predominantly found in animal‐based foods, which have a higher water footprint due to factors like feed crop irrigation and water usage in animal husbandry (Mekonnen and Hoekstra 2011, 2012). In a study examining the relationship between the environmental impact and nutritional content of sandwiches and beverages sold in a UK university cafeteria, the ecological impact score positively correlated with energy, saturated fat, vitamin B_12_, sodium, and iron (Graham et al. 2019). Ilhan et al. (2023) found that higher energy, protein, saturated fat, cholesterol, and iron intake increased diet‐related environmental factors. Heller et al. (2018) also presented that those individuals with higher daily energy intake had higher dietary carbon footprints. However, there are studies showing that energy density is not significantly associated with carbon footprints (Dahmani et al. 2022) and even that low energy density diets have a high carbon footprint (Tukker et al. 2011; Van Dooren et al. 2014). Generally, the type of food affects ecological footprints more than the energy density of food (González‐García et al. 2018). The primary sources of animal‐based protein, saturated fatty acids, cholesterol, vitamin B_12_, and iron are meat, poultry, dairy products, and eggs. Table salt, bread, other bakery products, meat, and fish are the main sodium sources (Blomhoff et al. 2023). Therefore, the positive correlation between animal‐based protein, saturated fat, cholesterol, vitamin B_12_, sodium, and iron with ecological footprint values in this study can be explained by the fact that university menus include more animal‐based foods, which are known to have higher harmful environmental impacts. Animal‐based food production is generally resource‐intensive, requiring more land, water, and energy, leading to a higher ecological footprint. It has been reported in many studies that menus rich in animal‐derived foods have a higher carbon footprint (Clune 2019; González‐García et al. 2020; Van De Kamp et al. 2018). In addition, many studies have underlined that plant‐based foods such as vegetables, fruits, grains, and legumes are responsible for a tiny part of the carbon footprints (Castañé and Antón 2017; González‐García et al. 2020). For this reason, it is stated that reducing the amount of animal‐based foods, especially red/processed meat dishes, on menus will reduce the dietary environmental burden (Benvenuti et al. 2022; Cleveland and Jay 2021; Van De Kamp et al. 2018). Replacing meat in the Dutch diet and consuming only foods with relatively low carbon footprints resulted in mean carbon footprint reductions varying from 28% to 46% (Van De Kamp et al. 2018). Benvenuti et al. (2022) reported that the carbon footprint of school menus can be reduced by 40% by choosing appropriate foods and restricting meat consumption. It has been noted that replacing beef with plant‐based foods will reduce the carbon footprint of a US university by 40% (Cleveland and Jay 2021). Lambrecht et al. (2023) estimated that replacing red meat with lower carbon footprint meat and red meat/poultry with fish or plant‐based proteins could reduce carbon footprints by 14%–46%. Similar to the carbon footprint, it is known that the water footprint is higher in animal‐based foods than plant‐based (Mekonnen and Hoekstra 2011, 2012). A study conducted in Spain reported that menus rich in animal‐derived foods led to a higher water footprint, primarily associated with beef (González‐García et al. 2020). Hatjiathanassiadou et al. (2019) showed that the water footprint of traditional menus was higher than that of vegetarian menus. Additionally, it has been determined that school menus that do not contain red meat reduce the carbon footprint by 32% and vegetarian menus by 54% compared to traditional menus (Cerutti et al. 2016). Moreover, the current study found that thiamine and zinc content were negatively associated with water footprint values. This may be related to the grain and legume content of university menus. The primary sources of thiamin are cereal products. Legumes, grains, and grain‐based products are rich dietary sources of zinc (Blomhoff et al. 2023). This result can be explained by the fact that university menus contain high amounts of cereal products and legumes with a relatively low water footprint (Mekonnen and Hoekstra 2011, 2012). In addition, our study showed that the ecological footprints were negatively related to the total fat (Tables 4 and 5). The reason for this may be the use of vegetable oils, especially sunflower oil, as the primary oil source in mass catering institutions. Vegetable oils have a lower water footprint than animal fats (Mekonnen and Hoekstra 2011, 2012).

Turkish cuisine is characterized by a high preference for foods of animal origin and low consumption of foods of plant origin, which may pose a sustainability challenge for the country (Ilhan et al. 2023). Generally, it is underlined that reducing the consumption of animal‐based foods and increasing the consumption of plant‐based foods can reduce the environmental burden and positively affect health by reducing the intake of saturated fatty acids (Castañé and Antón 2017). However, it is also emphasized that avoidance or lower intake of animal foods may contribute to the nutritional inadequacy of several micronutrients such as iron, calcium, and vitamin B_12_ (Van De Kamp et al. 2018). Therefore, a holistic approach should be considered, and recommendations to reduce the environmental footprint of food systems should be balanced with nutritional requirements for health. To improve university lunch menus' sustainability and nutritional quality, universities can implement several practical interventions. These may include reducing the portion sizes of animal‐based foods, especially red and processed meats, while increasing the availability of plant‐based proteins such as legumes and grains. Additionally, promoting the inclusion of more vegetables and fruits in menus can further reduce the ecological footprint and improve the nutritional value of meals. Moreover, reformulating university lunch menus to include more sustainable and nutritious options could significantly impact student and staff dietary behaviors. Providing a wider variety of plant‐based options might encourage them to make healthier choices, as exposure to such foods can shift their preferences over time. It has been shown that offering more nutritious, sustainable meal choices in university dining halls can influence students' and staff's long‐term eating habits (Seah et al. 2022). Therefore, reformulating menus to focus on sustainability and health could reduce the ecological footprints of university meals and support the development of more health‐conscious dietary behaviors in the student and staff population.

The current study has some strengths and limitations. Evaluating the nutritional content and ecological footprint of all accessible university menus in Türkiye and identifying regional differences are the strengths of this study. A limitation of the study is the use of international factors since there is currently no nationwide data on the carbon and water footprint values of foods in Türkiye. These international data may not accurately represent the ecological footprint of foods typically consumed in Türkiye, as regional dietary preferences and local food production methods can significantly differ. Therefore, there is a need for a national study in which carbon and water footprint values are calculated, considering geographical location, food preferences, and eating habits. In addition, the analysis of menus relied on secondary data sources, which may not always reflect the actual meals served at university dining halls in this study. Rather than relying solely on external data, primary environmental assessments would provide more accurate and context‐specific insights, enhancing the study's validity. Future studies could consider conducting primary environmental assessments using local data on food sourcing and production methods to better capture the real nutritional values and environmental impact of these meals. Another limitation is that vegan or vegetarian menus are not evaluated. Future studies evaluating these menus will allow a comparison of traditional and vegan/vegetarian university menus. One of the study's limitations is that it was conducted only on universities that publicize their online menus. At this point, changes may have been made at the last moment in the menus published online. Moreover, this study did not consider seasonality when evaluating university dining hall menus. Seasonal variations in ingredient availability and menu planning could influence the types of meals served, which may affect the generalizability of the findings. Additionally, excluding universities with missing menus or incomprehensible dish names may introduce selection bias and affect the sample's representativeness. Variations in menu naming conventions could have influenced data collection and interpretation. Future research could analyze menus from different seasons and establish standardized criteria for menu inclusion to minimize potential biases. The final limitation of this study is that the environmental effects of food waste should be considered. Direct observation for assessing dietary intake will help reveal the relationship between ecological footprint and food waste.

## 5 Conclusion

University dining halls are significant for encouraging adequate and balanced nutrition and establishing sustainable eating habits. This study concluded that Turkish universities needed nutritionally balanced menu planning due to menus' excessive energy and nutrient content. Given the importance of lunch as the main meal of the day, the nutritional profile of university lunch menus should be revisited to improve the diets of many young adults. When evaluating menus, it is essential to consider nutritional content and environmental effects together. This study observed that menus containing high‐energy and rich in animal‐based foods had a greater environmental impact. For this reason, balanced menus incorporating plant‐ and animal‐based foods are necessary to meet nutritional needs while reducing environmental harm. Increasing vegetables, fruits, legumes, nuts, and seeds while limiting red meat to a moderate level will enhance menu quality and sustainability. Besides, the complex interaction of factors such as students' and staff's cultural preferences, socioeconomic status, and food availability should also be considered during menu planning. Future research should explore how these factors influence individuals' acceptance of sustainable meal options. Understanding the attitudes of students and staff toward plant‐based and environmentally friendly menus will provide valuable insights for developing strategies to promote sustainable eating habits. Additionally, further studies should explore the cost‐effectiveness and feasibility of implementing sustainable menus in university settings. Integrating sustainability principles into national dietary guidelines is important. Policymakers should emphasize the importance of balanced menus that align with nutritional adequacy and environmental sustainability. Finally, considering the limitations of this study, including the reliance on international footprint factors, the use of external data, the exclusion of vegan/vegetarian menus, the lack of seasonality considerations, potential selection bias, and the absence of food waste analysis, future research should focus on conducting primary environmental assessments, incorporating local data, evaluating diverse menu types, and analyzing seasonal variations to provide a more comprehensive understanding of the nutritional and environmental impact of university dining hall meals.

## Author Contributions

**Ozge Yesildemir:** conceptualization, methodology, software, validation, formal analysis, data curation, writing – original draft preparation, writing – review and editing, and visualization.

## Conflicts of Interest

The author declares no conflicts of interest.

## Supporting information


Table S1.

Table S2.

## Data Availability

The data that support the findings of this study are available from the corresponding author upon reasonable request.
