# Supplementary material for: Energy and Nutritional Content of Lunch Menus in Turkish Universities: The Impact on Ecological Footprint
Source: Food Sci Nutr. 2025 Apr 8;13(4):e70149. doi: 10.1002/fsn3.70149 (PMC11976065; doi:10.1002/fsn3.70149)
Supplement: Supplementary file 1 — Table S1. [file FSN3-13-e70149-s002.docx]

**Table S1. Carbon footprint factors across food categories**

| **Foods** | **Carbon footprint factors (kg CO_2_ eq/kg)** |
| --- | --- |
| **Cereals** |  |
| Flour | 0.52 |
| Rice | 2.55 |
| Maize/corn | 0.58 |
| Wheat | 0.51 |
| Oats | 0.42 |
| Rye | 0.41 |
| Barley | 0.49 |
| Quinoa | 1.15 |
| **Fruits** |  |
| Melon | 0.24 |
| Apple | 0.36 |
| Pear | 0.42 |
| Watermelon | 0.32 |
| Dates | 0.35 |
| Orange | 0.37 |
| Kiwi | 0.38 |
| Grapes | 0.41 |
| Peach | 0.54 |
| Nectarine | 0.54 |
| Fig | 0.49 |
| Apricot | 0.62 |
| Tangerine | 0.46 |
| Pineapple | 0.50 |
| Grapefruit | 0.51 |
| Avocado | 1.30 |
| Sour cherry | 0.39 |
| Cherry | 0.39 |
| Quince | 0.31 |
| Strawberry | 0.58 |
| Raspberry | 0.84 |
| Cranberry | 0.92 |
| Blueberry | 0.92 |
| Olives | 0.63 |
| **Vegetables and Legumes** |  |
| Onion | 0.17 |
| Celery | 0.18 |
| Potato | 0.18 |
| Carrot | 0.22 |
| Cucumber/pickle | 0.23 |
| Beet | 0.23 |
| Pumpkin | 0.23 |
| Green beans | 0.51 |
| Mushroom | 0.27 |
| Turnip | 0.41 |
| Cauliflower | 0.38 |
| Broccoli | 0.38 |
| Tomato | 0.46 |
| Fennel | 0.72 |
| Artichoke | 0.48 |
| Black-eyed peas | 0.48 |
| Spinach | 0.54 |
| Garlic | 0.57 |
| Eggplant | 1.35 |
| Lentil | 1.03 |
| Asparagus | 0.83 |
| Lemon | 0.26 |
| Capsicums/Peppers | 0.66 |
| Lettuce | 3.70 |
| Peas | 0.60 |
| Red beans | 0.73 |
| Chickpeas | 0.77 |
| **Dairy Products** |  |
| Milk | 1.29 |
| Yoghurt | 1.43 |
| Cream | 5.85 |
| Cheese | 10.50 |
| Butter | 9.25 |
| **Meat Products** |  |
| Chicken meat | 3.15 |
| Turkey meat | 9.44 |
| Fish (all types) | 3.61 |
| Beef | 25.85 |
| Lamb meat | 20.61 |
| **Eggs** | 3.39 |
| **Nuts an Seeds** |  |
| Peanuts | 0.83 |
| Walnut | 1.53 |
| Pistachio | 1.53 |
| Almond | 1.54 |
| Chestnut | 0.62 |
| Hazelnut | 0.97 |
| Sesame | 0.88 |
| Sunflower seed | 1.41 |
| Coconut | 0.42 |

**References**

Clune, S., E., Crossin, and K., Verghese. 2017. Systematic review of greenhouse gas emissions for different fresh food categories. *Journal of Cleaner Production* 140: 766-783. https://doi.org/10.1016/j.jclepro.2016.04.082
