# Supplementary material for: Energy and Nutritional Content of Lunch Menus in Turkish Universities: The Impact on Ecological Footprint
Source: Food Sci Nutr. 2025 Apr 8;13(4):e70149. doi: 10.1002/fsn3.70149 (PMC11976065; doi:10.1002/fsn3.70149)
Supplement: Supplementary file 2 — Table S2. [file FSN3-13-e70149-s001.docx]

**Table S2. Water footprint factors across food categories**

| **Foods** | **Water Footprint Factor (m^3^/ton)** | **Green Water Footprint Factor (m^3^/ton)** | **Blue Water Footprint Factor (m^3^/ton)** | **Grey Water Footprint Factor**  **(m^3^/ton)** |
| --- | --- | --- | --- | --- |
| **Cereals** |  |  |  |  |
| Wheat flour | 1849 | 1292 | 347 | 210 |
| Wheat | 1826 | 1277 | 342 | 207 |
| Rice | 2230 | 1527 | 454 | 249 |
| Rice flour | 2628 | 1800 | 535 | 293 |
| Cracked corn | 1081 | 837 | 72 | 171 |
| Corn flour | 1253 | 971 | 83 | 199 |
| Corn starch | 1671 | 1295 | 111 | 265 |
| Wheat starch | 1436 | 1004 | 269 | 163 |
| Bread crumbs | 1608 | 1124 | 301 | 183 |
| Bread | 1608 | 1124 | 301 | 183 |
| Noodles | 1849 | 1292 | 347 | 210 |
| Pasta | 1849 | 1292 | 347 | 210 |
| **Fruits** |  |  |  |  |
| Fig | 3350 | 1527 | 1595 | 228 |
| Banana | 660 | 97 | 33 | 790 |
| Lemon | 642 | 432 | 152 | 58 |
| Plum | 2180 | 1570 | 188 | 422 |
| Dried apple | 6847 | 4678 | 1111 | 1058 |
| Pear | 922 | 645 | 94 | 183 |
| Raisin | 2433 | 1700 | 386 | 347 |
| Cherry | 1411 | 1098 | 213 | 99 |
| Peach | 910 | 583 | 188 | 139 |
| Apple | 822 | 561 | 133 | 127 |
| Grapes | 608 | 425 | 97 | 87 |
| Tangerine | 748 | 479 | 118 | 152 |
| Watermelon | 235 | 147 | 25 | 63 |
| Cherry | 1604 | 961 | 531 | 112 |
| Apricot | 1287 | 694 | 502 | 92 |
| Strawberry | 347 | 201 | 109 | 37 |
| Raspberry | 413 | 293 | 53 | 67 |
| Orange | 560 | 401 | 110 | 49 |
| Orange juice | 1018 | 729 | 199 | 90 |
| Currant | 499 | 457 | 19 | 23 |
| Blueberry | 845 | 341 | 334 | 170 |
| Cranberry | 276 | 91 | 108 | 77 |
| Dates | 2277 | 930 | 1250 | 98 |
| Coconut | 2449 | 2433 | 2 | 15 |
| **Vegetables and Legumes** | | | | |
| Okra | 576 | 474 | 36 | 65 |
| Cabbage | 280 | 181 | 26 | 73 |
| Tomato | 214 | 108 | 63 | 43 |
| Cucumber | 353 | 206 | 42 | 105 |
| Potato | 287 | 191 | 33 | 63 |
| Onion | 345 | 192 | 88 | 65 |
| Green onion | 272 | 176 | 44 | 51 |
| Carrot | 195 | 106 | 28 | 61 |
| Green pepper | 379 | 240 | 42 | 97 |
| Eggplant | 362 | 234 | 33 | 95 |
| Garlic | 589 | 337 | 81 | 170 |
| Lettuce | 237 | 133 | 28 | 77 |
| Green beans | 561 | 320 | 54 | 188 |
| Spinach | 292 | 118 | 14 | 160 |
| Cauliflower | 285 | 189 | 21 | 75 |
| Broccoli | 285 | 189 | 21 | 75 |
| Brussels sprouts | 285 | 189 | 21 | 75 |
| Zucchini | 336 | 228 | 24 | 84 |
| Artichoke | 818 | 478 | 242 | 98 |
| Asparagus | 2150 | 1524 | 119 | 507 |
| Tomato paste | 855 | 431 | 253 | 171 |
| Ketchup | 534 | 270 | 158 | 107 |
| Lentil | 5874 | 4324 | 489 | 1060 |
| Dried beans | 5053 | 3945 | 125 | 983 |
| Kidney beans | 5053 | 3945 | 125 | 983 |
| Chickpeas | 4177 | 2972 | 224 | 981 |
| Corn | 1081 | 837 | 72 | 171 |
| Peas | 595 | 382 | 63 | 150 |
| **Dairy Products** |  |  |  |  |
| Milk | 1020 | 863 | 86 | 72 |
| White cheese | 5060 | 4264 | 439 | 357 |
| Kashar cheese | 5060 | 4264 | 439 | 357 |
| **Meat Products** |  |  |  |  |
| Chicken meat | 4325 | 3545 | 313 | 467 |
| Lamb meat | 8763-10412 | 9813 | 522 | 76 |
| Beef | 15415 | 14414 | 550 | 451 |
| Minced meat | 15415 | 14414 | 550 | 451 |
| **Eggs** | 3265 | 2592 | 244 | 429 |
| **Nuts and Seeds** |  |  |  |  |
| Walnut | 9280 | 5293 | 2451 | 1536 |
| Pistachio | 11363 | 3095 | 7602 | 666 |
| Hazelnut | 10515 | 7627 | 2180 | 709 |
| Almond | 16095 | 9264 | 3816 | 3015 |
| Chestnut | 2750 | 2432 | 174 | 144 |
| Peanut | 3974 | 3526 | 214 | 234 |
| Poppy | 2188 | 1723 | 0 | 464 |
| Sesame | 9371 | 8460 | 509 | 403 |
| **Sugar** | 1782 | 1184 | 487 | 111 |
| **Fats** |  |  |  |  |
| Butter/Margarine | 5553 | 4695 | 465 | 393 |
| Olive oil | 14431 | 11826 | 2388 | 217 |
| Olive | 3015 | 2470 | 499 | 45 |
| Sunflower oil | 6792 | 6088 | 299 | 405 |
| **Spices** |  |  |  |  |
| Mint | 288 | 206 | 63 | 19 |
| Red pepper | 7365 | 5869 | 1125 | 371 |
| Black Pepper | 7611 | 6540 | 467 | 604 |
| Cinnamon | 15526 | 14853 | 41 | 632 |
| Clove | 61205 | 59834 | 30 | 1341 |
| Coriander, Fennel | 8280 | 5369 | 1865 | 1046 |
| Ginger | 1657 | 1525 | 40 | 92 |
| **Others** |  |  |  |  |
| Vanilla | 126505 | 86392 | 39048 | 1065 |
| Coffee | 18925 | 18153 | 139 | 633 |
| Cocoa | 15636 | 15492 | 3 | 141 |
| Chocolate | 17196 | 16805 | 198 | 193 |

**References**

Mekonnen, M.M., and A.Y. Hoekstra. 2011. The green, blue and grey water footprint of crops and derived crop products. *Hydrology and Earth System Sciences* 15(5): 1577-1600. https://doi.org/10.5194/hess-15-1577-2011

Mekonnen, M.M., and A.Y. Hoekstra. 2012. A global assessment of the water footprint of farm animal products. *Ecosystems* 15(3): 401-415. https://doi.org/10.1007/s10021-011-9517-8
